# Finite mixture clustering of human tissues with different levels of IGF-1 splice variants mRNA transcripts

**DOI:** 10.1186/s12859-015-0689-7

**Published:** 2015-09-15

**Authors:** Michele Pelosi, Marco Alfò, Francesca Martella, Elisa Pappalardo, Antonio Musarò

**Affiliations:** 1 Institute Pasteur Cenci-Bolognetti, DAHFMO-Unit of Histology and Medical Embryology, IIM, Sapienza University of Rome, Via A. Scarpa 16, 00161 Rome, Italy; 2 Dipartimento di Scienze Statistiche, Sapienza University of Rome, P. le A. Moro 5, 00185 Rome, Italy; 3 Sir William Dunn School of Pathology, University of Oxford, South Parks Road, Oxford, OX1 3RF UK; 4 Center for Life Nano Science@Sapienza, Istituto Italiano di Tecnologia, Viale Regina Elena 291, Rome, 00161 Italy

**Keywords:** Finite mixture model, Finite mixture of linear mixed models, IGF-1, Alternative splicing, Multiple alternatively spliced mRNA, Tissue profiling, Biostatistics, Gene expression levels

## Abstract

**Background:**

This study addresses a recurrent biological problem, that is to define a formal clustering structure for a set of tissues on the basis of the relative abundance of multiple alternatively spliced isoforms mRNAs generated by the same gene. To this aim, we have used a model-based clustering approach, based on a finite mixture of multivariate Gaussian densities. However, given we had more technical replicates from the same tissue for each quantitative measurement, we also employed a finite mixture of linear mixed models, with tissue-specific random effects.

**Results:**

A panel of human tissues was analysed through quantitative real-time PCR methods, to quantify the relative amount of mRNA encoding different IGF-1 alternative splicing variants. After an appropriate, preliminary, equalization of the quantitative data, we provided an estimate of the distribution of the observed concentrations for the different IGF-1 mRNA splice variants in the cohort of tissues by employing suitable kernel density estimators. We observed that the analysed IGF-1 mRNA splice variants were characterized by multimodal distributions, which could be interpreted as describing the presence of several sub-population, i.e. potential tissue clusters. In this context, a formal clustering approach based on a finite mixture model (FMM) with Gaussian components is proposed. Due to the presence of potential dependence between the technical replicates (originated by repeated quantitative measurements of the same mRNA splice isoform in the same tissue) we have also employed the finite mixture of linear mixed models (FMLMM), which allowed to take into account this kind of within-tissue dependence.

**Conclusions:**

The FMM and the FMLMM provided a convenient yet formal setting for a model-based clustering of the human tissues in sub-populations, characterized by homogeneous values of concentrations of the mRNAs for one or multiple IGF-1 alternative splicing isoforms.

The proposed approaches can be applied to any cohort of tissues expressing several alternatively spliced mRNAs generated by the same gene, and can overcome the limitations of clustering methods based on simple comparisons between splice isoform expression levels.

## Background

Alternative splicing, which can be detected in more than 90 % of multiexon genes [1], is considered a leading process giving rise to cellular and tissues diversity in higher eucariotes [2]. It has been estimated that there are, on average, at least seven alternative splicing events per multiexon human gene [1]; considering that each tissue can potentially express a peculiar set of splice isoforms, it is easy to deduce that splicing complexity can profoundly contribute to tissue diversity.

In this study, we employed a finite mixture model (FMM) to cluster a cohort of tissues on the basis of the relative abundance of multiple alternatively spliced mRNAs. We show how this clustering approach, based on a probabilistic model, can overcome the limitations of clustering methods based on simple comparisons between splice isoform expression levels.

FMM have been used for several decades in the analysis of high-dimensional complex data, and are now experiencing a progressive popularity, due to the increase in the computing power [3, 4]. The classical areas of application for FMMs are social sciences and economics, but several applications of FMMs are extending to genetics, biology, natural sciences, psychology, medicine [4].

FMMs are probabilistic models that can be applied to complex data whenever the experimental observations are drawn from several subpopulations which are not known *a priori* [5]. While the clustering context is certainly a major area of application, FMMs may also be employed to produce a semiparametric (kernel-type) estimate for a multimodal distribution, with the feature of being more parsimonious than standard kernel-based estimators [5]. For this reason, FMMs can be fruitfully employed also in the presence of high dimensional data.

In this manuscript we aim at solving a recurrent problem in molecular biology that is to define an appropriate clustering structure for a set of tissues that display differential concentrations in multiple alternatively spliced mRNA variants generated by the transcription of the same gene. To better explain how a suitable solution to this problem can be found, we describe the analysis of a paradigmatic case, where we aim at clustering a cohort of human tissues on the basis of the relative abundance of IGF-1 mRNA splicing variants. However, since the data measurement consist in three technical replicates for each observed splice isoform in each observed tissue, a dependence issue may arise, and standard finite mixture models for clustering cannot be the appropriate choice. For this reason, in this study we have also employed the method described by Celeux et al. [6] to cluster tissues in presence of replicated data.

**Fig. 1:**
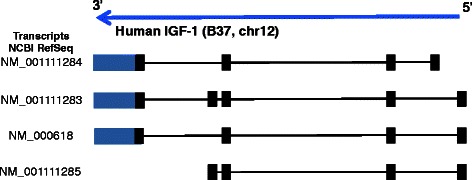
Schematic representations of the human IGF-1 NCBI RefSeq transcripts. A schematization of human IGF-1 gene locus (on chromosome 12) is also shown. Exons (black rectangles), introns (black lines), and UTRs (blue rectangles) size are not proportional to the real size of the corresponding portions of the IGF-1 transcripts. The correct proportion of the transcripts can be visualized at University of California Santa Cruz Genome Browser Gateway (https://genome-euro.ucsc.edu/cgi-bin/hgGateway). Refer to “Methods” section to deduce which transcripts were detected by each specific TaqMan assay used in the quantitative real-time PCR experiments

In human tissues, the transcription from a single IGF-1 gene can generate multiple splicing isoforms (summarized in Fig. 1; for an exhaustive review see [7–9]) which encode multiple proteins, comprising variable amino- and carboxy-terminal amino acid sequences.

In particular, splicing in the 3′-region of the IGF-1 transcripts give rise to different mRNA splicing variants (Fig. 1) and as a consequence, this generates an important molecular variability in the carboxy-terminus of the mature IGF-1 peptide that represents the so-called “E-peptide” [10]. The different E-peptides might modulate the biological actions, the stability or the bioavailability of the IGF-1 protein [10, 11]. For this reason, different IGF-1 mRNA splicing variants could be preferentially abundant in some tissues, and scarce or even absent in other.

In spite of the great importance of IGF-1 in physiology and pathology [8, 11, 12], no comprehensive quantitative studies or detailed analyses of the relative abundance of IGF-1 splice isoforms mRNAs in panels of human tissues have been reported so far.

In human, alternative splicing in the 3′-region of the IGF-1 transcripts give rise to three different mRNA splicing variants: IGF-1 Ea, IGF-1 Eb and IGF-1 Ec (Fig. 1) [11, 13]. IGF-1 is bona fide expressed by all human tissues; however, it remains unknown whether all human tissues are also able to splice all the three splice variants Ea, Eb and Ec.

IGF-1 Ea is considered the “standard isoform” expressed locally in muscle [12, 13], and the predominant splicing variant in liver [14]; for this reason, this particular isoform is often referred to as a “local muscle specific” IGF-1 isoform (mIGF-1) or “muscle/liver type” isoform [14–16]. The isoform IGF-1 Ec has been detected in muscles subjected to damage or exercise, and it is often defined as a “mechano growth factor” (MGF) [14–16]. However it is not clear whether IGF-1 Ec is also significantly spliced in non-muscular tissues or its presence can alter the relative balance of the two other splicing isoforms IGF-1 Ea and IGF-1 Eb.

To gain a better understanding of IGF-1 alternative splicing, we performed a quantitative analysis of the IGF-1 mRNA splice variants in human tissues. In a panel of human tissues, after a preliminary data equalization (which allows us to compare relative mRNAs levels of the different IGF-1 variants across the different tissues), an exploratory quantitative analysis on IGF-1 mRNA splice variants was performed. This analysis showed that IGF-1 Ea, Eb and Ec were spliced in all human tissues, and that their concentrations is characterized by multimodal distributions. This was clearly appreciated by employing kernel density estimation to smooth the observed empirical frequency distributions.

We then proposed a finite mixture model (FMM) with multivariate Gaussian kernel to formally cluster human tissues into subgroups with homogeneous values of concentration for the IGF-1 splice variants. Since this model-based clustering approach does not allow to consider repeated measurements, and since the analysed quantitative data contains technical replicates for each measurement, we have also applied the method of Finite mixture of linear mixed models (FMLMM) by Celeux et al. [6], which has been defined to produce a finite mixture clustering of repeated measurements. The two proposed approaches show substantial similarities in the obtained results, with a clear cut biological interpretation.

The approaches presented in this work can be regarded as a general framework for the formal clustering of any cohort of tissues (or cells) expressing alternatively spliced mRNAs, and can overcome the limitations of clustering methods based on the simple comparison between splice variants levels.

## Results and discussion

### Data pre-processing and exploratory analysis on IGF-1 splice variants concentration in human tissues

A panel of 20 human tissues was analysed through quantitative real-time PCR, using specific IGF-1 splicing isoforms assays, to detect the mRNAs encoding IGF-1 Ea, IGF-1 Eb and IGF-1 Ec splice variants (Fig. 1).

**Fig. 2:**
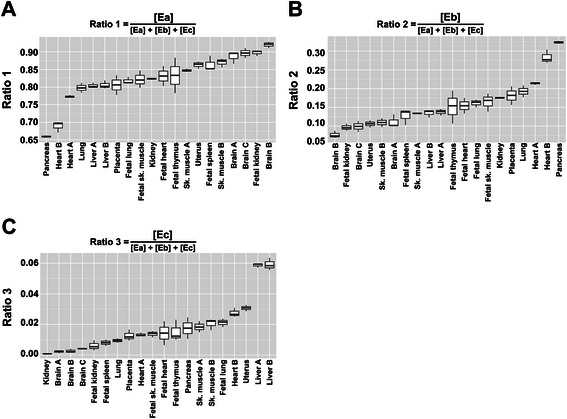
Boxplots of the relative abundance of IGF-1 splice variants mRNA in a cohort of 20 human tissues. **a** IGF-1 Ea compositional ratio values; **b** IGF-1 Eb compositional ratio values; **c** IGF-1 Ec compositional ratio values. The bottom and top of each box represent the first and third quartile, whereas the band inside the box represents the median. The whiskers represent the minimum and maximum of the distributions. Tissues are ordered according increasing mean values

Data derived from real-time PCR analysis (the comparative C_T_ method was used to obtain a quantitative measure of each IGF-1 splice variant mRNA, see Methods) were equalized by means of the “compositional ratios” (or “concentration ratios”, see Methods), referred to as Ratio 1, Ratio 2, Ratio 3, as shown in Table 1 and Fig. 2. This transformation allows to compare the relative mRNA concentrations for the IGF-1 splicing isoforms across the different tissues in the panel. However, it also results in permanent loss of the original amplitude (magnitude) in the expression levels of each isoform in each tissue.

**Table 1:** Descriptive statistics for the human IGF-1 splice variants mRNA compositional ratios

	Min.	1st quartile	Median	Mean	3rd quartile	Max.
Ratio 1 (IGF-1 Ea)	0.6560	0.8000	0.8335	0.8276	0.8733	0.9293
Ratio 2 (IGF-1 Eb)	0.06916	0.10820	0.13910	0.15380	0.17750	0.32690
Ratio 3 (IGF-1 Ec)	0.000721	0.007274	0.014130	0.018560	0.023310	0.063630

Ratio 1 $=\frac{\left[Ea\right]}{\left[Ea\right]+\left[Eb\right]+\left[Ec\right]}$; Ratio 2 $=\frac{\left[Eb\right]}{\left[Ea\right]+\left[Eb\right]+\left[Ec\right]}$; Ratio 3 $=\frac{\left[Ec\right]}{\left[Ea\right]+\left[Eb\right]+\left[Ec\right]}$

Descriptive statistics for the observed human IGF-1 splice variants mRNA compositional ratios in the cohort of 20 different human tissues. Minimum, maximum, 1st quartile, median, 3rd quartile and mean are shown for each compositional ratio. Compositional ratios are defined in the lowest part of the table

Additionally, the formal constraint [Ea]+[Eb]+[Ec]=1 holds, i.e. the sum of the relative concentrations for the three IGF-1 splicing isoforms is constant (and equal to one) in all the analysed tissues. The constraint is not only a simple theoretical assumption: in this respect, we used a IGF1_PAN_ specific TaqMan assay that simultaneously detects all the human IGF-1 isoforms (see Methods), and we performed preliminary *ad hoc* tests to verify the null hypothesis that [IGF1_PAN_]= [Ea]+[Eb]+[Ec]; our empirical findings suggested that we have not enough empirical evidence to reject the null hypothesis (data not shown). In other words, according to experimental observations, in each human tissue the sum of relative concentrations for the three mRNA IGF-1 splicing variants Ea, Eb and Ec, is not substantially different from the total IGF-1 mRNA pool, and this result is consistent with the theoretical assumptions.

A preliminary exploratory data analysis was performed on the equalized data; we report some summary statistics for the compositional ratios in Table 1. We then summarized the distribution of the concentration ratios, by looking at the within-tissues variability, as shown by the boxplots in Fig. 2a, b and c, where the observed tissues are ordered by increasing mean values.

According to the findings in Table 1 and Fig. 2, we could deduce the following:

i) all the human tissues we have analysed are able to splice the three IGF-1 mRNA isoforms Ea, Eb and Ec;
ii) all human tissues in the cohort can splice, on a relative scale, a greater amount of the variant Ea, a smaller amount of the variant Eb, and a minimal amount of the variant Ec.

Therefore, it emerges a general concept that should be taken into consideration when we perform a quantitative analysis of mRNA splice variants: when we refer to “high values” or “low values” of expression for one specific splice mRNA variant, it is always relatively to the values of concentration for the other splice variants in the same tissue or in a specific cohort of tissues. For example, in our specific cohort, high values of concentration for the isoform Eb will be always lower to Ea, even with respect to low values of concentration for isoform Ea (this can be evinced by looking at Table 1). Also, in our cohort, high values of concentration for the isoform Ec will be lower to Eb, even with respect to low values of concentration for isoform Eb (Table 1).

The boxplots in Fig. 2 do not provide a formal clustering of tissues. However, they are very interesting from a general observational perspective, and they can suggest which are the tissues showing more marked expression levels for a specific splice variant. For example, the tissues [Liver A and Liver B] seem to contain high concentrations of variant Ec, while the tissues [Brain A, Brain B and Brain C] seem to contain low concentrations of variant Ec, relatively to the cohort of all the observed tissues (Fig. 2c).

### Kernel density estimates of the distribution of IGF-1 splicing isoforms mRNA concentrations in human tissues

**Fig. 3:**
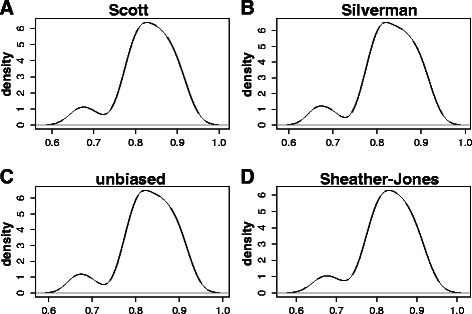
Kernel density estimates of the distribution of IGF-1 Ea splice variant mRNA compositional ratios in a cohort of 20 different human tissues. IGF-1 Ea splice variant mRNA compositional ratio (Ratio 1) is defined in Fig. 2 and in Table 1. Bandwidth selection: **a** Scott rule of thumb, **b** Silverman rule of thumb, **c** unbiased cross-validation, **d** Sheather-Jones plug-in

**Fig. 4:**
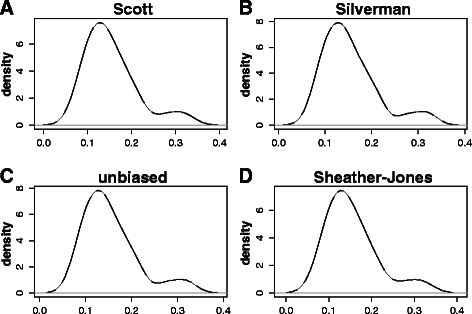
Kernel density estimates of the distribution of IGF-1 Eb splice variant mRNA compositional ratios in a cohort of 20 different human tissues. IGF-1 Eb splice variant mRNA compositional ratio (Ratio 2) is defined in Fig. 2 and in Table 1. Bandwidth selection: **a** Scott rule of thumb, **b** Silverman rule of thumb, **c** unbiased cross-validation, **d** Sheather-Jones plug-in

**Fig. 5:**
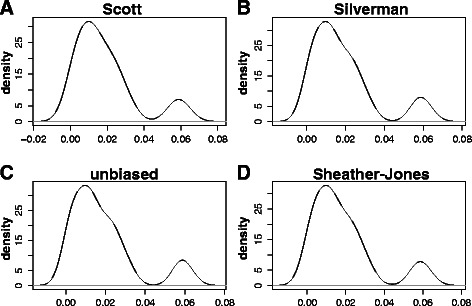
Kernel density estimates of the distribution of IGF-1 Ec splice variant mRNA compositional ratios in a cohort of 20 different human tissues. IGF-1 Ec splice variant mRNA compositional ratio (Ratio 3) is defined in Fig. 2 and in Table 1. Bandwidth selection: **a** Scott rule of thumb, **b** Silverman rule of thumb, **c** unbiased cross-validation, **d** Sheather-Jones plug-in

We employed kernel density estimates (KDE) to get a further step in the characterization of the distribution of the observed values for the three IGF-1 splicing variants Ea, Eb and Ec mRNA concentrations in the cohort of human tissues (Figs. 3, 4 and 5).

The quality of a kernel estimate heavily depends on the value of the bandwidth [17]. By using a small value, we may have a very local (i.e. extremely variable) estimate, while a too high value may result in over-smoothing. For this reason, we have employed different choices for the bandwidth. In Figs. 3, 4 and 5, that correspond to KDE for the variants Ea, Eb and Ec, respectively, panel A refers to the Scott rule of thumb [18], panel B to the Silverman rule of thumb [17], panel C to the unbiased cross-validation based bandwidth [19], while panel D refers to the Sheather and Jones plug-in method [20] (see also the section Methods and Refs. [21] and [22] for a critical discussion on density estimation).

As it can be easily evinced by looking at Figs. 3, 4 and 5, the kernel density estimates suggest the presence of at least two subpopulations in each of the analysed IGF-1 splicing variants, thus resulting in multimodal density estimates (Figs. 3, 4 and 5).

To be more specific, with respect to the human splice isoform IGF-1 Ea, we may observe that at least two tissue subpopulations can be recognized in the analysed cohort: the first one has higher frequency and, likely, represents what we can roughly call the “IGF-1 Ea mRNA standard population”, while the second one is more on the left side of the range of the observed values and shows a lower frequency (Fig. 3). On the basis of the boxplot shown in Fig. 2, we may speculate that the latter subpopulation likely includes those tissues having a concentration of the splice isoform IGF-1 Ea lower than the average.

Opposite conclusions can be inferred by looking at the kernel density estimates for variants IGF-1 Eb and IGF-1 Ec, which are displayed in Figs. 4 and 5.

Some asymmetries in the higher frequency kernel component for the expression levels of the splice isoforms IGF-1 Ea and IGF-1 Ec can also be easily noticed (Figs. 3 and 5). These asymmetries may indicate either the presence of a third subpopulation, not clearly distinguishable from the high-frequency component, or, rather, a departure from symmetry of the population-specific density (Figs. 3 and 5).

However, as we have already remarked on the grounds of Fig. 2, the empirical evidence we obtain from Figs. 3, 4 and 5 is mainly observational, and cannot lead to any formal inference on the existence of tissue subgroups that show different values for the observed concentration of a given IGF-1 splice variants.

### Finite mixture models to classify tissues with different levels of IGF-1 splicing isoforms

As presented in the previous section, in human tissues the analysed levels of IGF-1 mRNA splice variants Ea, Eb and Ec are characterized by multimodal distributions, which could be interpreted as being composed by several sub-populations.

In this context the finite mixture models (FMM) with Gaussian kernel could provide a formal, convenient, model-based method to cluster the cohort of human tissues in several, but potentially overlapping, subpopulations with homogeneous values of mRNA concentration for one or more IGF-1 splicing isoform. For the empirical analysis, we employed simultaneously all the technical replicates (usually triplicates, sometimes duplicates when the experimental triplicates were not available) for each human IGF-1 mRNA splice isoform (Ea, Eb and Ec), in each different tissue (*X*_irk_, see the Methods section).

According to the FMM, the population of tissues is divided in G components (i.e. in 2 or more “subpopulations”, or “clusters”, or “subgroups”) [5]; each component is graphically identified by an elliptical shape, which usually represents a (multivariate) Gaussian density; in this case, given the unit constraint, we have fitted the model to the couple (Ea, Eb), so that the cluster-specific distribution we are considering is a bivariate Gaussian density.

The clusters (components of the finite mixture) are thus characterized by a center, defined by the corresponding mean value, and by the elliptical contour, summarized by the covariance matrix. By definition, the latter may be parameterized as a function of 3 parameter sets, which describe scale, shape and direction, respectively [23]. Each of these parameter sets may be constant, variable or, whenever appropriate, independent, leading to 10 different association structures for each choice of the number of components [23].

To choose the appropriate number of components (that is clusters) in the population, the model was fitted for a progressively increasing number of clusters (G = 2, …,10) for each of the different structures for the cluster-specific covariance matrix. The solution corresponding to the lowest Bayesian Information Criterion score (BIC score) [24] has been retained. In our analysis the best model (i.e. the model with the lowest BIC score) corresponds to G = 3 components with an association structure defined by the acronym “EEV”, standing for Equal scale, Equal shape and Variable orientation across the components [25].

After fitting the model, we may define a formal clustering of the tissues by allocating each tissue to the cluster with the highest probability of component membership, see Methods. This procedure is often referred to as MAP (maximum a posteriori) clustering.

**Fig. 6:**
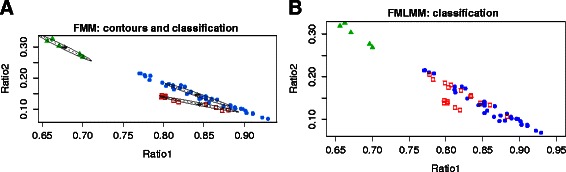
Plots of the observed IGF-1 values. **a** contours and classification obtained through the FMM (mclust) and **b** classification obtained through the FMLMM. Legend: blue dots: component #1; red squares: component #2; green triangles: component #3. Ratio 1 and Ratio 2 are defined in Fig. 2 and in Table 1

In our FMM there is a clear distinction in the center of the distribution for clusters [#1 and #2] when compared to cluster #3 (Table 2, and Fig. 6a). Components #1 and #2 are both characterized by a higher concentration of isoform IGF-1 Ea when compared to component #3 (Table 2); the latter component is characterized by a relatively higher concentration of the isoform IGF-1 Eb, even if it should be observed that in any tissue the concentration of the isoform IGF-1 Eb is lower when compared to the concentration of the isoform IGF-1 Ea (as already discussed, see Tables 1 and 2).

**Table 2:** Finite Mixture Model (FMM): parameter estimates

	Component #1	Component #2	Component #3
μ_(IGF1-Ea)k_	0.8420	0.8443	0.6772
μ_(IGF1-Eb)k_	0.1468	0.1166	0.2992
σ^2^_(IGF1-Ea)k_	0.0016	0.0023	0.0011
σ^2^_(IGF1-Eb)k_	0.0013	0.0006	0.0018
σ_(Ea, Eb)k_	−0.0014	−0.0011	−0.0014
ρ_(Ea, Eb)k_	−0,9884	−0,9827	−0,9878
π_k_	0.68322399	0.22616579	0.09061023

Finite Mixture Model (FMM): parameter estimates for the component specific means (μ), variances/covariances (σ), correlations (ρ) and prior probabilities (π)

Components #1 and #2 differ mainly as a function of the orientation of the ellipse, that is the orientation of the corresponding covariance matrix estimates, showing a different correlation between the observed values for IGF-1 Ea and IGF-1 Eb, which is higher (in absolute value) for component #1 with respect to component #2 (Table 2, and Fig. 6a).

We assigned each tissue to a cluster by using a MAP criterion, as introduced above; the resulting classification with an estimate of the classification entropy, is shown in Table 3. It is worth considering that by allocating each tissue replicate to a specific component, we force the value of the posterior probability (which ranges from 0 to 1) to be 0 or 1 and, therefore, we insert some approximation error in our model. For some tissue replicate, this produces a higher variability due to the uncertainty in the allocation, which is higher when the tissue replicate is characterized by values of posterior probability of component membership that are very similar across the different components.

**Table 3:** Finite Mixture Model (FMM): maximum a posteriori allocation

Component # tissue	1	2	3	Entropy (average)
Brain A	3	0	0	0.0204
Brain B	3	0	0	0.3287
Brain C	2	0	0	0.1691
Fetal heart	2	0	0	0.0000
Fetal kidney	3	0	0	0.1690
Fetal lung	3	0	0	0.0000
Fetal sk. muscle	3	0	0	0.0000
Fetal spleen	3	0	0	0.0199
Fetal thymus	3	0	0	0.0150
Heart A	3	0	0	0.0000
Heart B	0	0	3	0.0067
Kidney	2	0	0	0.0000
Liver A	0	3	0	0.0008
Liver B	0	3	0	0.0005
Lung	2	0	0	0.0000
Pancreas	0	0	2	0.0001
Placenta	3	0	0	0.0000
Sk. muscle A	3	0	0	0.0010
Sk. muscle B	2	1	0	0.1928
Uterus	0	3	0	0.0035

Finite Mixture Model (FMM): posterior classification of human tissues into components. The table shows the maximum a posteriori allocation (MAP) of each tissue of the panel to the highest probability component of the finite mixture. The associated Entropy (average) is also shown

To give an exemplification on how uncertainty is generated by allocation of tissue replicates to specific components, let us assume we have two components, namely cluster A and cluster B, and that according to MAP allocation we can assign a tissue replicate to cluster A. Suppose now the posterior probabilities for the tissue are equal to 0.99 for cluster A and 0.01 for cluster B, respectively. In this case, the tissue can be allocated to cluster A with a very low uncertainty. However, if the posterior probabilities are equal to 0.51 and 0.49 for clusters A and B, respectively, the tissue can still be allocated to cluster A, but the uncertainty of the classification is higher.

In this work, model-based clustering has been performed using the mclust library [25] developed for the open-source software R; in this context, classification uncertainty is measured by the Shannon entropy. Table 3 also describes the average uncertainty in the MAP classification for the human tissues data (see the column referring to entropy in Table 3). A higher classification uncertainty can therefore be associated to points located between components #2 and #3 when compared to points located between components #1 and #3. Looking in more details at these points, we observe that the highest values of uncertainty are associated to the following tissues: Brain B, Brain C, Fetal kidney and Skeletal muscle B, and range between 0.16 and 0.32 (with a maximum value of 1).

As shown in Table 3 each tissue was allocated to the specific component with a score of 3 replicates out of 3 (or 2 out of 2 if the third technical replicate was not available for experimental reasons). Only the Skeletal muscle B (which, as discussed, was characterized by a relatively high allocation uncertainty) was associated to a partial allocation discrepancy: in fact, two experimental replicates were assigned to component #1, whereas the remaining technical replicate was assigned to the component #2 (Table 3).

### Finite mixture of linear mixed models to classify tissues with different levels of IGF-1 splicing isoforms accounting for dependence between replicates

As we have noticed in the previous section, using FMM to analyze quantitative real-time PCR data was very effective, and only in one case a technical replicate was not assigned by the FMM to the same component as the other two replicates (Table 3).

FMM clustering approach is based on considering the technical replicates (originated by repeated quantitative measurements of the concentration of same mRNA splice isoform in the same tissue) as independent units, increasing the sample size. To take into account potential dependence between technical replicates we adopted a further clustering strategy, i.e. the finite mixture of linear mixed models (FMLMM) [6]. This method, developed by Celeux et al. [6] can be considered as a particular specification of the method proposed by Basford and McLachlan [26] for the analysis of three way data (in this case, tissues by isoforms by replicates). However, application of FMLMM to cluster tissues in presence of replicated data required a specific implementation (mclust does not allow for this extension) and generated some less favourable effects: i) a reduced sample size (in fact the replicates were not treated as statistical independent units anymore) and, ii) each individual (tissue-specific) sequence became a KR-dimensional sequence, where K denotes the number of splice variants and R the number of replicates. Consequently, this reduced the number of possible models to be fitted, due to joint increase in the number of parameters and the decrease in the sample size.

According to FMLMM, the population of tissues is divided into G components; each component is now identified by a regression model with a component-specific intercept (playing the role of overall mean), a tissue-specific effect that accounts for technical variability and dependence between replicates from the same tissue [6]. Altogether these different sources of variation define an elliptical shape, which represents a (multivariate) Gaussian density in a higher dimension (2 by R, where R is the number of replicates), with all the sources of variation (included the measurement error) concurring to define the covariance matrix (see the Methods section).

To choose the appropriate number of components (that is the appropriate number of clusters) in the population, we have considered the solution corresponding to the lowest Bayesian Information Criterion score (BIC score) [24]. However, it should be noted that given the reduced sample size, and given the highly parameterized model specification, we had the chance to estimate the model only for G = 2,3 and the best model, i.e. the model with the lowest BIC corresponded to G = 3 components. As described in the previous paragraph for FMM, after fitting the model with FMLMM, we may define a MAP clustering of the tissues by allocating each tissue to the cluster with the highest probability of component membership (see Methods).

As for FMM, FMLMM shows a clear distinction in the centers of the distributions corresponding to clusters [#1 and #2] when compared to cluster #3 (Tables 4-5 and Fig. 6b). The latter component is characterized by a relatively higher concentration of the isoform IGF-1 Eb (Table 4), even if it should be observed (as already discussed, see Tables 1 and 2) that in any tissue the concentration of the isoform IGF-1 Eb is lower when compared to the concentration of the isoform IGF-1 Ea. In the context of FMLMM, however, the difference between components #1 and #2 is not due to a difference in the orientation of the covariance matrix as seen for the FMM (Table 2 and Table 5). Rather, the two components #1 and #2 seem to share the same orientation, with higher values in the second component (Table 4 and Fig. 6b).

**Table 4:** Finite Mixture of Linear Mixed Models (FMLMM): parameter estimates I

	Component #1	Component #2	Component #3
μ_(IGF1-Ea)k_	0.8171	0.8629	0.6742
μ_(IGF1-Eb)k_	0.1546	0.1262	0.3033
σ^2^_(IGF1-Ea)k_	0.00059	0.00014	0.00010
σ^2^_(IGF1-Eb)k_	0.00046	0.00014	0.00014
π_k_	0.3229	0.5772	0.0999

Finite Mixture of Linear Mixed Models (FMLMM): parameter estimates for the component specific means (μ), variances/covariances (σ), and prior probabilities (π)

**Table 5:** Finite Mixture of Linear Mixed Models (FMLMM): parameter estimates II

1st component	IGF1Ea	IGF1Eb
IGF1Ea	0.00006	−0.00008
IGF1Eb	−0.00008	0.00016
2nd component		
IGF1Ea	0.00182	−0.00176
IGF1Eb	−0.00176	0.00171
3rd component		
IGF1Ea	0.00021	−0.00028
IGF1Eb	−0.00028	0.00038

Finite Mixture of Linear Mixed Models (FMLMM) parameter estimates: covariance matrices for the individual-specific random effects

We assigned each tissue to a cluster, by using a MAP criterion as introduced above; the resulting classification is shown in Table 6, with an estimate of the classification entropy. For some tissue, the allocation produces a higher variability due to the uncertainty in the allocation, which is higher when the tissue presents similar values for posterior probability across the different components. Looking in more details at these points, we observe that in FMLMM the highest values of uncertainty are associated to the following tissues: Fetal lung, Fetal skeletal Muscle, Kidney, Lung. These values are not consistent with the entropy values we have derived from the application of FMM (Table 3). Moreover, the very low entropies we could often observe in FMLMM (Table 6) are partially due to the higher number of parameters respect to the sample size. Fig. 6a and b shows the partition of tissues obtained adopting the FMM and the FMLMM, respectively. Table 7 reports the same information under the shape of a confusion matrix.

**Table 6:** Finite Mixture of Linear Mixed Models (FMLMM): maximum a posteriori allocation

Tissue	Component	Entropy
Brain A	2	0.0000
Brain B	2	0.0000
Brain C	2	0.0000
Fetal heart	1	0.0000
Fetal kidney	2	0.0000
Fetal lung	2	0.6804
Fetal sk. muscle	1	0.1189
Fetal spleen	2	0.0097
Fetal thymus	1	0.0000
Heart A	2	0.0000
Heart B	3	0.0102
Kidney	2	0.1573
Liver A	1	0.0041
Liver B	1	0.0002
Lung	2	0.1039
Pancreas	3	0.0000
Placenta	1	0.0311
Sk. muscle A	2	0.0501
Sk. muscle B	2	0.0001
Uterus	2	0.0001

Finite Mixture of Linear Mixed Models (FMLMM): posterior classification of human tissues into components. The table shows the maximum a posteriori allocation (MAP) of each tissue of the panel to the highest probability component of the finite mixture. The associated Entropy is also shown

**Table 7:** Confusion matrix

Tissue count	Cluster FMLMM			
Cluster FMM	#1	#2	#3	Total
#1	4	10		14
#2	2	2		4
#3			2	2
Total	6	12	2	20

Confusion matrix: clustering obtained via FMM (rows) by clustering obtained via FMLMM (columns)

In our perspective, for the resolution of this specific biological problem the mclust library [25] still represents the methods of choice: is a straightforward analysis tool which can be easily handled by practitioners, geneticists and biological researchers. Additionally, though more appropriate clustering methods might be proposed for the case of replicated measurements, FMM still remains, at least to our opinion, a quite reliable and flexible approach.

### A comprehensive analysis of the abundance of IGF-1 mRNA splice variants in human tissues

From the FMM posterior classification listed in Table 3 we could finally infer a formal clustering structure for the human tissues, based on the relative abundance of the mRNAs for the IGF-1 alternative splicing isoforms.

As shown in Table 3, each tissue was allocated to the specific component with a score of 3 replicates out of 3, or 2 out of 2 when the third technical replicate was not available for experimental reasons (a recurring circumstance in real-time PCR measures). Only the Skeletal muscle B (which, as discussed previously, was characterized by a relatively high allocation uncertainty) was associated to a partial allocation discrepancy: in fact, two experimental replicates were assigned to component #1, whereas the remaining technical replicate was assigned to the adjacent component #2. However, this may be explained by all three replicates being on the tails of the corresponding distributions (Fig. 6a).

The majority of the human tissues in the panel were allocated by FMM as belonging to component #1 (Table 3). Components #1 and #2 are characterized by a higher concentration of the isoform IGF-1 Ea with respect to component #3 (Fig. 6a and Table 2). As remarked above, components #1 and #2 differ mainly as a function of the orientation of the ellipse, with a different correlation between the observed values for IGF-1 Ea and IGF-1 Eb (Table 2). Liver A and Liver B (mRNA derived from two independent groups of donors) and Uterus were assigned to component #2 (Fig. 6a and Table 2). Remarkably, we observed from the boxplots in Fig. 2c that Liver A, Liver B and Uterus are characterized by the highest relative concentration of IGF-1 Ec. Therefore we can speculate that the component #2 is characterized by a peculiar relatively higher concentration of the isoform IGF-1 Ec.

The tissues allocated by FMM to component #3 are characterized by a relatively higher concentration of the isoform IGF-1 Eb, when compared to components #1 and #2, i.e. Pancreas and Heart B (Fig. 2b and Table 2).

It is interesting to observe that most of the results of the allocation are consistent when we consider the same tissue, but pools of RNA collected from independent groups of donors: for example Brain A, Brain B and Brain C are all allocated to component #1; Liver A and Liver B are both allocated to component #2. However Heart A and Heart B are assigned to two highly separated subgroups, i.e. to components #1 and #3, respectively. This means that pools of RNA from the same tissue, but collected from different individuals (in which tissues are possibly subjected to different patho-physiological circumstances) may sometimes exhibit radically different IGF-1 splice isoforms balance. It has been reported, for example, that IGF-1 Ec increased during post-infarcted myocardium remodelling [27].

To better understand the rationale behind the clusterization obtained by means of FMM, we consider tissues Brain A, Brain B and Brain C, which are three pools of RNAs from three independent groups of human donor individuals. Looking at the boxplots in Fig. 2 we may roughly derive that these tissues are characterized by “a low concentration of IGF-1 Ec”. However, such cluster does not formally exist, since, due to the unit constraint, we have considered Ea and Eb only. Therefore, Brain A, Brain B and Brain C are formally allocated to component #1, that is a cluster characterized by a high concentration of isoform IGF-1 Ea and, in particular, by a relatively lower concentration of IGF-1 Eb and by a relatively lower concentration of IFG-1 Ec.

We can summarize the results of our FMM study on human tissues as follows:

- all the human tissues in the panel were able to splice the isoforms IGF-1 Ea, IGF-1 Eb and IGF-1 Ec.
- the fulfilment of the formal equation [Ea]+[Eb]+[Ec]=1 and the compositional ratios we have calculated have helped to compare the relative mRNA concentrations for the different IGF-1 splice variants within each single tissue and across the different tissues.
- in all the human tissues the relationship [Ea] > [Eb] > [Ec] holds, at least as an empirical evidence. Specifically, every tissue showed a lower concentration of the isoform IGF-1 Eb when compared to IGF-1 Ea; at the same time, in every tissue the concentration of the isoform IGF-1 Ec was lower than the concentration of the isoform IGF-1 Eb.
- by defining and applying a FMM we have been able to formally cluster the analyzed tissues into three subgroups: i) tissues with relatively high concentration of IGF-1 Ea and relatively lower concentration of IGF-1 Ec (Cluster # 1); ii) tissues with relatively high concentration of IGF-1 Ea and IGF-1 Ec (Cluster # 2); iii) tissues with relatively high concentration of IGF-1 Eb (Cluster # 3).

As discussed so far, we implemented FMLMM as a further clustering strategy to take into account the potential dependence between technical replicates. When considering the clustering results from this latter approach, we observed that the separation between [component #1, component #2] and [component #3] is still well retained, and this result is consistent with FMM (Fig. 6). The peculiar difference of FMLMM when compared with FMM is the relative composition of component #1 and component #2: while in the FMM the prior probabilities (π) for component #1 and component #2 were, respectively, 0.68 and 0.22 (Table 2), for the FMLMM the corresponding prior probabilities were 0.32 and 0.57 (Table 4), with six tissues moving from one component to the other (Tables 3 and 6).

Additionally most of the results of the allocation for FMLMM are consistent when we consider pools of RNA collected from independent human donors: for example Brain A, Brain B and Brain C were all allocated by FMLMM to component #2; Liver A and Liver B were both allocated to component #1; Skeletal muscle A and B were both allocated to component #2 (Table 6).

To better understand the rationale associated with the FMLMM clustering, we can observe that:

- the tissues allocated by FMLMM to component #3 (Pancreas and Heart B) are characterized by a peculiar relatively high concentration of the isoform IGF-1 Eb when compared to tissues allocated in the components #1 and #2 (Fig. 2 and Table 6).
- Liver A and Liver B were allocated by FMLMM to component #1, and show an average concentration of isoform IGF-1 Ea and Eb, and a particular high concentration of IFG-1 Ec (Fig. 2 and Table 6). However, the other sample units belonging to components #1 cannot be clearly distinguished from tissues allocated to component #2; in this respect, the FMLMM approach is, at least to some extent, less efficient than FMM, where the three components were appreciably better characterized.

Roughly speaking, FMLMM component #1 and component #2 may be considered as describing two different tails of the same distribution, the only relevant difference being represented by Liver A and Liver B, and this poses the question of whether we definitively need for FMLMM a 3 component solution, or we can rather use a 2 component solution.

Further statistical validations and biological experiments are needed to assess unequivocally whether components #1 and #2 belong from two distinct clusters (as suggested by FMM) or belong to one single cluster.

## Conclusions

In this study we propose two approaches to address the general biological problem of clustering a group of tissues on the basis of the relative abundance of multiple spliced mRNA, generated by the transcription of the same gene. We present a paradigmatic case, the quantitative analysis by real-time PCR of IGF-1 mRNA splice variants in human tissues.

The starting point of this study is the appropriate preliminary equalization of the quantitative data, followed by summary statistics and non-parametric estimators based on the observed distributions (kernel density estimates).

The clustering approach proposed in this work is based on a finite mixture model (FMM) with Gaussian components, which is an efficient model based clustering approach, available in several open-source statistical computer packages.

In order to take into account potential dependence between technical replicates from the same tissue, we propose a further clustering strategy, the finite mixture of linear mixed models (FMLMM), which required a specific implementation.

In our analysis, both FMM and FMLMM provided a convenient yet formal setting for model-based clustering of a cohort of human tissues expressing three spliced mRNAs. These approaches can be applied by the biologist to any cohort of tissues expressing several alternatively spliced mRNAs, and can overcome the limitations of classification methods based on the simple comparisons between splice variants expression levels.

## Methods

### Human cDNAs and RNAs

All the human samples, namely cDNA and RNAs, used in this study have been purchased from Clontech and Ambion; according the suppliers specifications, all human cDNAs and RNA samples used were obtained from normal donors, essentially free from diseases. Pancreas cDNA (derived from total RNA pooled from 15 male/female Caucasians individuals), heart (A) cDNA (whole heart) (derived from total RNA from 3 male Caucasians), lung cDNA (derived from total RNA from 1 male Caucasians), liver (A) cDNA (derived from total RNA from 1 male Caucasians), placenta cDNA (derived from total RNA from 11 female Caucasians), kidney cDNA (derived from total RNA from 4 male/female Caucasians), skeletal muscle (A) cDNA (derived from total RNA from 4 male/female Caucasians), brain (A) cDNA (whole brain) (derived from total RNA from 4 male Caucasians) were all purchased from Clontech (Human MTC Panel I cDNAs). Heart (B) cDNA (whole heart) was generated by Reverse Transcription (RT) (see below) from human heart total RNA (RNA from Clontech, pooled from 3 male Caucasians); liver (B) cDNA was generated by RT from human liver total RNA (RNA from Clontech, pooled from 3 Asian males); skeletal muscle (B) cDNA was generated by RT from human skeletal muscle total RNA (RNA from Clontech, pooled from 1 Caucasian male); uterus cDNA was generated by RT from human uterus total RNA (RNA from Clontech, pooled from 8 female Caucasians); brain (B) cDNA (whole brain) was generated by RT from brain total RNA (RNA from Clontech, pooled from 4 male Asians); Brain (C) cDNA (whole brain) was generated by RT from human brain RNA (FirstChoiche human brain total RNA from Ambion, from 1 male Caucasian). Fetal lung cDNA, Fetal skeletal muscle cDNA, Fetal heart cDNA, Fetal thymus cDNA, Fetal spleen cDNA, Fetal kidney cDNA, were all generated by RT from human fetal total RNA purchased from Clontech.

#### Reverse Transcription (RT)

First-strand cDNAs from each total RNA was generated using a Reverse Transcription kit with random primers (High Capacity cDNA Reverse Transcription kit; Applied Biosystems) starting from 1 μg of total RNA as described in [28].

#### Real-time PCR

Newly synthesized cDNAs were diluted 5-fold in DNase-free water and 5 % of the cDNA (or an equivalent quantity of the purchased cDNAs) was then used in each real-time PCR assay. Each single determination was performed in technical triplicate, using a 7500 Fast Real-Time PCR (Applied Biosystems), and the TaqMan Universal Master Mix II (Applied Biosystems).

The comparative C_T_ method was used to make a quantitative measure of each IGF-1 splice variant and of the housekeeping gene mRNA. In order to assure a valid ∆∆C_T_ calculation, only empirically validated TaqMan assay with an experimental efficiency approaching 100 % were used. For each assay, the efficiency of the reference gene (GAPDH) amplification and the efficiency of each IGF-1 splice variant amplification, were experimentally determined and compared within a progressive template dilution. The validation experiments for each TaqMan assay were performed according the general recommendation described in the Applied Biosystems User Bulletin (ABI Prism 7700 Sequence Detection System #2) and each assay was considered validated when the efficiency of the housekeeping gene amplification and the target gene amplification were approximately equal.

#### TaqMan assays

All the TaqMan assays were purchased from Applied Biosystems (ABI). Refer to Fig. 1 to deduce the exon organization of the different splice isoforms and the corresponding NCBI RefSeq transcripts detected by each TaqMan assay used. **Human, IGF-1 “PAN”** TaqMan assay (ABI Hs01547656_m1) able to detect the human IGF-1 transcripts corresponding to NCBI RefSeq NM_001111284, NM_001111283, NM_000618, NM_001111285. **Human, IGF-1 Ea** TaqMan assay (ABI Hs01547657_m1) able to detect the human IGF-1 transcripts corresponding to NCBI RefSeq. NM_001111284, NM_000618. **Human, IGF-1 Eb** TaqMan assay (ABI Hs01555481_m1) able to detect the human IGF-1 transcripts corresponding to NCBI RefSeq NM_001111283, NM_001111285. **Human IGF-1 Ec** TaqMan assay (ABI Hs03986524_m1) able to detect the human IGF-1 transcript corresponding to NCBI RefSeq NM_ NM_001111283. **Human GAPDH** TaqMan assay (ABI Hs02758991_g1): reference (housekeeping) gene able to detect the transcripts for human glyceraldehyde-3-phosphate dehydrogenase (NCBI reference sequence of the transcripts detected by the assay: NM_002046, NM_001256799).

### Statistical analysis

All statistical analyses, including graphical representation, summary statistics, exploratory statistics, kernel density estimation, finite mixture clustering and finite mixture of linear mixed models have been performed using the open source software R, release 3.0.1 (codename: “Good sport”).

### Kernel density estimation

The kernel density estimation (KDE) of a density function refers to non-parametric approaches to estimate the probability density function of random variable X based on the empirical distirbution of an observed sample, say **x** = (*x*_1_, …, *x*_*n*_), whose elements are assumed to be independent and identically distributed accoding to an unknown density *f*(⋅|*θ*). The aim is at providing an estimate of *f* by using the information stored in the observed sample **x**. The kernel density estimator for *f* is defined by:

$$ {\widehat{f}}_h\left(\kern0.1em x\right)=\frac{1}{n}{\displaystyle \sum_{i=1}^n{K}_h\left(x-{x}_i\right)=}\frac{1}{nh}{\displaystyle \sum_{i=1}^nK\left(\frac{x-{x}_i}{h}\right)} $$

where *K*_*h*_(⋅) is the kernel, that is a non-negative function that integrates to one, defined over a zero-mean random variable, and *h* is a parameter, often referred to as the *bandwidth*, that controls the smoothing of the data. Intuitevely, one may choose a small h to provide the best fit to the observed data; however, there is always a trade-off between the bias of the estimator and its variance. By using a small bandwidth, we may have a very local (i.e. extremely variable) estimate, while adopting a too high value may result in over-smoothing the observed distribution. For this reason, in the present analysis, we used 4 different bandwidths, based on the Scott [18] and Silverman [17] rules of thumb, on unbiased cross-validation, see Bowman [19] and on the Sheather & Jones plug-in [20], respectively. See Sheather [22] for a comparison of the methods, refere to Sheather [21] for a thourough discussion on density estimation. A range of kernel can be used; due to its conveninet mathematical properties, the Gaussian kernel is often used; in the present context, we have used the Gaussian and the Epanechnikov kernels, but for sake of space and given the estimates do not substantially difffer, we report only those based on Gaussian kernel.

### Finite mixture of multivariate Gaussian densities

To provide a formal clustering of human tissues in this study, we have decide to adopt a two-step strategy: first, we have used a finite mixture model with Gaussian components to cluster tissues in homogeneous classes, considering the technical replicates for each tissue as independent. Then, we have used a finite mixture of linear mixed-effects models, see Celeux et al. [6] and Ng et al. [29] to take into account of possible clusters in the sample while allowing for dependence between technical replicates corresponding to the same tissue. We will provide a brief description of both methods below.

### Model-based clustering via finite mixtures

We assume that each tissue belong to one of *G* discrete classes, say *P*_*g*_, *g* = 1, …, *G*, that is the population of tissues is partitioned in *G* disjoint classes, *P* = ∪ _*g* = 1_^*G*^ *P*_*g*_. Tissues belonging to a given class have a class-specific density or probability mass function; the density function *f*(⋅) is of the same parametric form across classes, but is parameterized by a class-specific parameter vector, say *θ*_*g*_, *g* = 1, …, *G*. Clearly, we may extend this approach allowing for the class-specific densities to have a class-specific parametric form, but this is quite well beyond the scope of the present work. Finite mixture models are given the choice of a suitable density function *f*(⋅), and for a given splice isoform *l* = 1, 2, the finite mixture model specifies the marginal density for a set of *r* = 1,.., *R* replicates referring to the *i*-th tissue as follows:

$$ f\left({\mathbf{x}}_i\right)={\displaystyle \sum_g}{\pi}_gf\left({\mathbf{x}}_i\Big|{\theta}_g\right)={\displaystyle \sum_g}{\pi}_g{\displaystyle \prod_r}f\left({\mathbf{x}}_{ir}\Big|{\theta}_g\right) $$

leading to the following observed data likelihood:

$$ L\left(\pi, \theta \right)={\displaystyle \prod_{i=1}^n{\displaystyle \sum_g}{\pi}_gf\left({\mathbf{x}}_i\Big|{\theta}_g\right)={\displaystyle \prod_{i=1}^n{\displaystyle \sum_g}{\pi}_g{\displaystyle \prod_r}f\left({\mathbf{x}}_{ir}\Big|{\theta}_g\right)}} $$

which can be considered as a specific version of the model-based clustering approach introduced by Basford and McLachlan (1985) for three-way data [26]. Here, the terms *π*_*g*_, *g* = 1, …, *G* represent the prior probability that a tissue belongs to the *g*-th class, *g* = 1, …, *G*, while the vector **x**_*i*_ includes the set of measures technical replicates **x**_*ir*_, *r* = 1, …, *R*, and, for each replicate, the values for the splice isoforms, *l* = 1,2. The assumption that:

$$ f\left({\mathbf{x}}_i\Big|{\theta}_g\right)={\displaystyle \prod_r}f\left({\mathbf{x}}_{ir}\Big|{\theta}_g\right) $$

mimics the *local independence* assumption; it says that, conditional on belonging to the class *P*_*g*_ the replicates corresponding to the same tissue are independent. However, due to the lack of general software routines to estimate the former model, we have further assumed that the technical replicates from the same tissues are independent. This oviously simplifies the analysis and allows the use of standard software, as it will beclear in the following. In this case, the likelihood reduce to

$$ L\left(\pi, \theta \right)={\displaystyle \prod_{i=1}^n{\displaystyle \prod_{r=1}^R{\displaystyle \sum_g}{\pi}_gf\left({\mathbf{x}}_{ir}\Big|{\theta}_g\right)}} $$

This assumption has also the result that the sample size is increased from *n* to *nR*. We can postulate the existence of a set of binary indicator variables, defined as:

$$ {z}_{irg}=\left\{\begin{array}{l}1\ \mathrm{if}\ \left(i,r\right)\in {P}_g\hfill \\ {}0\ \mathrm{else}\hfill \end{array}\right. $$

indicating the component a tissue replicate belongs to. Should these be observed, the estimation problem would simply reduce to a multi-class problem, where the class-specific parameter *θ*_*g*_ is to be estimated only on tissues belonging to the *g*-th component, *g* = 1, …, *G*. However, the indicator variables are unobserved, and the number *G* of components is also unknown and must be estimated together with other model parameters. Thus, in the following, we will refer to the couple (**x**_*ir*_, **z**_*ir*_) as the complete data, and base our inferences on the following procedure. Assuming that the *G*-dimensional (latent) variable **z**_*ir*_ has a multinomial distribution:

$$ f\left({\mathbf{z}}_{ir}\right)={\displaystyle \prod_{g=1}^G}{\pi}_g^{z_{irg}} $$

we derive the following density for the *complete* data:

$$ f\left({\mathbf{x}}_{ir},{\mathbf{z}}_{ir}\right)={\displaystyle \prod_{g=1}^G}{\left[f\left({\mathbf{x}}_{ir}\Big|{\theta}_g\right){\pi}_g\right]}^{z_{irg}} $$

Assuming that the tissue and the replicates are independent on each other, we have the following expression for the likelihood function:

$$ L\left(\theta \kern0.1em ,\pi \right)={\displaystyle \prod_{i=1}^n}{\displaystyle \prod_{r=1}^R\left\{f\left({x}_i,{z}_i\right)\right\}}={\displaystyle \prod_{i=1}^n}{\displaystyle \prod_{r=1}^R\left\{{\displaystyle \prod_{g=1}^G}{\left[f\left({\mathbf{x}}_{ir}\Big|\theta \right){\pi}_g\right]}^{z_{irg}}\right\}} $$

Therefore, the log-likelihood function for the complete data can be written as:

1 $$ {\ell}_c\left(\kern0.1em \theta \kern0.1em ,\pi \right)\propto {\displaystyle \sum_i}{\displaystyle \sum_{g=1}^G}{z}_{irg}\left[ \log \left({\pi}_g\right)+ \log {f}_{irg}\right], $$

where *f*_*irg*_ = *f*(**x**_*ir*_|*θ*_*g*_ ). Since the latent vector **z**_*ig*_ is unobservable, we use the EM algorithm to perform maximum likelihood estimation;this is an iterative algorithm, where two steps can be identified. In the first step, the E-step, of the algorithm, we define the log-likelihood for *observed* data by taking the expectation of the log-likelihood for *complete* data over the unobservable class indicator vector **z**_*ir*_ given the observed data **x**_*ir*_ and the current maximum likelihood estimates of model parameters, say *θ* ^(*t*)^. In other words, at the *t*-th step, we replace *z*_*ig*_ by its conditional expectation:

2 $$ {\widehat{z}}_{irg}\left({\theta}^{(t)}\right)={w}_{irg}^{(t)}=\frac{\pi_g^{(t)}{f}_{irg}\left(\cdot \Big|{\theta}^{(t)}\right)}{{\displaystyle \sum_{g=1}^G}{\pi}_g^{(t)}{f}_{irg}\left(\cdot \Big|{\theta}^{(t)}\right)} $$

which represents the posterior probability that the *ir*-th tissue replicate belongs to the *g*-th component, given the observed data. The conditional expectation of the complete log-likelihood given the observed data **x** is expressed by the function:

3 $$ {Q}^{(t)}\left(\theta, \pi \right)={E}_{\theta}^{(t)}\left\{{\ell}_c\left(\kern0.1em \theta, \pi \right)\Big|x\right\}\propto {\displaystyle \sum_i}{\displaystyle \sum_r{\displaystyle \sum_{g=1}^G}{w}_{irg}^{(t)}\left\{ \log {f}_{irg}\left(\cdot \Big|{\theta}^{(t)}\right)+ \log \left({\pi}_g^{(t)}\right)\right\}} $$

If we use, a multivariate Gaussian density with component-specific parameter set given by *θ*_*g*_ = (**μ**_*g*_, **Σ**_*g*_) as class-specific density, we obtain the following expression:

4 $$ {Q}^{(t)}\left(\theta, \pi \right)\propto {\displaystyle \sum_i}{\displaystyle \sum_r{\displaystyle \sum_{g=1}^G}{w}_{ig}^{(t)}\left\{-\frac{1}{2} \log \left(\left|{\varSigma}_g\right|\right)-\frac{1}{2}{\left[{\mathbf{x}}_{ir}-{\boldsymbol{\upmu}}_g^{(t)}\right]}^T{\varSigma}_g^{-1}\left[{\mathbf{x}}_{ir}-{\boldsymbol{\upmu}}_g^{(t)}\right]+ \log \left[{\pi}_g^{(t)}\right]\right\}} $$

where the superscript *T* denote matrix transpose, and under the constraint ∑_*g*_ *π*_*g*_ = 1. In the M-step of the algorithm, we maximize *Q* ^(*t*)^(⋅) with respect to *θ*_*g*_, and obtain the following ML estimates for the parameters of the Gaussian density in the *g*-th component:

5 $$ {\widehat{\boldsymbol{\upmu}}}_g^{(t)}=\frac{{\displaystyle \sum_{i,r}}{w}_{irg}^{(t)}{\mathbf{x}}_{ir}}{{\displaystyle \sum_{i,r}}{w}_{irg}^{(t)}},\kern1em {\widehat{\Sigma}}_g^{(t)}=\frac{{\displaystyle \sum_{i,r}}{w}_{irg}^{(t)}{\left[{\mathbf{x}}_{ir}-{\widehat{\boldsymbol{\upmu}}}_g^{(t)}\right]}^T\left[{\mathbf{x}}_{ir}-{\widehat{\boldsymbol{\upmu}}}_g^{(t)}\right]}{{\displaystyle \sum_{i,r}}{w}_{irg}^{(t)}};\kern1em g=1,\dots, G $$

while te prior estimates are:

6 $$ {\widehat{\pi}}_g^{(t)}=\frac{{\displaystyle \sum_{i,r}}{w}_{irg}^{(t)}}{n} $$

which are well known results from ML in finite mixtures. Solving these equations for given weights *w*_*irg*_^(*t*)^ and updating the weights for given parameter estimates *θ* ^(*t*)^ defines an EM algorithm. The E and M steps are alternated repeatedly until convergence, which is obtained with a sequence of log-likelihood values which is bounded from above. Thus, we reach a solution for a given *G*, which can be used to estimate model parameters when we move to the solution for *G* + 1 classes. The corresponding solutions may be compared using penalized likelihood criteria, such as AIC o BIC, since the standard conditions do not holt to use the standard LRT statistic (the corresponding distribution under the null hypothesis is not the standard one). Once the EM algorithm has reached its end, the tissue replicate can be allocated to the comonent having the highest posterior probability *w*_*irg*_^(*t*)^; in this way we obtain the clustering we are looking for, and compare estimated parameter values corresponding to the different classes, to characterize the obtained results. To avoid the well know sensitivity of the results obtained by the EM algorithm from the choice of the starting values for model parameters and/or posterior probabilities, we employed, prior to the EM algorithm, a short-length CEM algorithm [30] which is quite flexible and robust to the presence of extreme values. This procedure often outperforms the one based on the comparison (in terms of penalized likelihood criteria) of results obtained using several random starts for the basic EM algorithm.

### Finite mixtures of linear mixed-effects models

As previously stated, in order to improve the quality of the clustering results, we should take into account the technical variability deriving from the replicated measurements taken on the same tissue. In this case, linear mixed-effects models (LMMs) are defined to model repeated measures for a given statistical unit (in our case human tissue), and allow to separate measurement error variation from between-units variation (variability of the same splice variant between different tissues) and within-units variation (between technical replicates from the same tissue). To propose a model-based clustering tool for technical replicates, the standard finite mixture model (FMM) previously discussed, has to be modified to the LMMs context. The idea was developed by Celeux et al. [6], where a mixture of LMMs is proposed in order to account for the variability in measurements while performing clustering.

In details, let **x**_*i*_ be the observed 2*R*-dimensional vector, representing the values for the *i*-th tissue, corresponding to the *R* technical replicated measurements on the 2 splice variants. To take into account replicated measurements in the finite mixture framework, Celeux et al. [6] simply added the assumption that the replicated measurements of a statistical unit (in our case tissue) belong to the same mixture component. In this way, we are able to model the covariance structure between the *r*-th and *r* '-th technical replicate on the *i*-th tissue from the *l*-th splice isoform, cov(*x*_*ilr*_, *x*_*ilr* '_). Thus, it is assumed that the observed 2*R*-dimensional vectors **x**_1_, …, **x**_*n*_ are drawn from a mixture of *G* components with unknown proportions *π*_1_, …, *π*_*G*_ (∑_*g*_ *π*_*g*_ = 1), and that, conditional on belonging to the *g*-th component of the mixture, each vector **x**_*i*_ referring to the *i*-th tissue can be specified by the following linear mixed-effect model:

7 $$ {\mathbf{x}}_i=\mathbf{V}\left({\boldsymbol{\upalpha}}_g+{\mathbf{u}}_{ig}\right)+{\mathbf{e}}_{ig} $$

where **V** is a known (2*R* × 2) design matrix defined by

8 $$ \mathbf{V}=\left(\begin{array}{cc}\hfill {\mathbf{1}}_R\hfill & \hfill {\mathbf{0}}_R\hfill \\ {}\hfill {\mathbf{0}}_R\hfill & \hfill {\mathbf{1}}_R\hfill \end{array}\right) $$

with **1**_*R*_ and **0**_*R*_ representing unit and null vectors of size *R*, **α**_*g*_ is a 2-dimensional vector of fixed effects modelling the conditional mean of **x**_*i*_ in the *g*-th component, **u**_*ig*_ represents a zero-mean 2-dimensional vector of random effects shared by the replicates corresponding to the same tissue capturing the technical variability, while **e**_*ig*_ represents the measurement error vector. The random effects **u**_*ig*_ and the measurement error vectors **e**_*ig*_ are assumed to be mutually independent. Furthermore, the distributions of **e**_*ig*_ are and **u**_*ig*_ are assumed to be multivariate Gaussian, MVN(**0**, **Ω**_*g*_) and MVN(**0**, **Φ**_*g*_), respectively.

It is worth to notice that Celeux et al. [6] specified the covariance matrix **Φ**_*g*_ as diagonal; however, in our study, we have considered a non-diagonal covariance matrix since we allow for correlation between measurements corresponding to different splice variants on the same tissue. Moreover, Celeux et al. [6] specified the covariance matrix **Ω**_*g*_ to be equal to *σ*_*g*_^2^ **I**_2*R*_, with **I**_2*R*_ being a 2*R* × 2*R* identity matrix; in order to allow the *g*-th component-variance to be different between the 2 splice variants, we assumed that **Ω**_*g*_ = *diag*(**Vσ**_*g*_^2^), where **σ**_*g*_^2^ = (*σ*_*g*1_^2^, *σ*_*g*2_^2^)'.

Given these assumptions, the component-specific mean vector and covariance matrix of **x**_*i*_ are

$$ {\boldsymbol{\upmu}}_g=\mathbf{V}{\boldsymbol{\upalpha}}_g $$

and

$$ {\boldsymbol{\Sigma}}_g=\mathbf{V}{\boldsymbol{\Phi}}_g\mathbf{V}\hbox{'}+{\boldsymbol{\Omega}}_g, $$

respectively. In other words, the model does not only assumes dependence between two technical replicates (on the same tissue and for the same splice variant), but also between measurements corresponding to different splice variants. Ng et al. [29] further extended this model, adding another random effect which induces dependency among statistical units from the same class.

The estimation of the model parameters of a finite mixture of linear mixed-effects model can be obtained by maximum likelihood (ML) via the EM algorithm. The missing data are of two types: the indicator variables **z**_*i*_ indicating the component a tissue belongs to and the randon effects **u**_*ig*_ (*i* = 1, …, *n*) for each class.

Let *π* = (*π*_1_, …, *π*_*G*_) be the mixing proportions, *θ*_*g*_ = (**α**_*g*_, **Φ**_*g*_, **σ**_*g*_^2^) be the parameter vector associated to each component *P*_*g*_ *g* = 1, …, *G*, and *θ* = (**α**_1_, …, **α**_*g*_, **Φ**_1_, …, **Φ**_*G*_, **σ**_1_^2^, …, **σ**_*G*_^2^). The log-likelihood associated to the complete data (**x**, **z**) is given by

9 $$ {\ell}_c\left(\kern0.1em \theta, \pi \Big|{\mathbf{x}}_i,{\mathbf{z}}_i,{\mathbf{u}}_{ig}\right)={\displaystyle \sum_i}{\displaystyle \sum_{g=1}^G}{z}_{ig} \log \left({\pi}_gf\left({\mathbf{x}}_i,{\mathbf{u}}_{ig}\Big|{\theta}_g\right)\right) $$

Conditionally on the *g-th* component, **x**_*i*_ is a realization from a MVN(**α**_*g*_ **V**, **VΦ**_*g*_ **V** '), therefore, *f*(**x**_*i*_, **u**_*ig*_|*θ*_*g*_) is a Gaussian distribution with mean $$ {\boldsymbol{\upmu}}_g=\left(\begin{array}{c}\hfill {\boldsymbol{\upalpha}}_g\mathbf{V}\hfill \\ {}\hfill \mathbf{0}\hfill \end{array}\right) $$ and covariance matrix $$ {\sum}_g=\left(\begin{array}{ll}\mathbf{V}{\boldsymbol{\Phi}}_g\mathbf{V}\hbox{'}+{\boldsymbol{\Omega}}_g\hfill & \mathbf{V}{\boldsymbol{\Phi}}_g\hfill \\ {}{\boldsymbol{\Phi}}_g\mathbf{V}\hbox{'}\hfill & {\boldsymbol{\Phi}}_g\hfill \end{array}\right). $$

At the *t*-th iteration, the E step, we compute the expectation of the complete-data log-likelihood conditional on the observed data and the current value of the parameter *θ* ^(*t*)^, *π* ^(*t*)^ which is given by

10 $$ \begin{array}{ll}{Q}^{(t)}\left(\kern0.1em \theta \kern0.1em ,\pi \right)=E\left\{{\ell}_c\left(\kern0.1em \theta \kern0.1em ,\pi \Big|{\mathbf{x}}_i,{\mathbf{z}}_i,{\mathbf{u}}_{ig}\right)\Big|\mathbf{x},{\theta}^{(t)},{\pi}^{(t)}\right\}\hfill & ={\displaystyle \sum_i}{\displaystyle \sum_{g=1}^G}{w}_{ig}^{(t)} \log \left({\pi}_g^{(t)}\right)\hfill \\ {}\hfill & +{\displaystyle \sum_i}{\displaystyle \sum_{g=1}^G}{w}_{ig}^{(t)}E\left\{f\left({\theta}_g\Big|{\mathbf{x}}_i,{\mathbf{u}}_{ig}\right)\Big|\mathbf{x},{\theta}^{(t)}\right\}\hfill \end{array} $$

with

11 $$ \begin{array}{ll}f\left({\theta}_g\Big|{\mathbf{x}}_i,{\mathbf{u}}_{ig}\right)=\hfill & -\frac{1}{2}\left(2R+2\right) \log \left(2\pi \right)-\frac{1}{2}2R \log \left(\left|{\boldsymbol{\Omega}}_g\right|\right)\\&\qquad\qquad\;- \log \left(\left|{\boldsymbol{\varphi}}_g\right|\right)\hfill \\ & -\frac{1}{2}\left({\mathbf{x}}_i-\mathbf{V}\left({\boldsymbol{\alpha}}_g+{\mathbf{u}}_{ig}\right)\right)^{\prime}{{\boldsymbol{\Omega}}}_g^{-1}\left({\mathbf{x}}_i-\mathbf{V}\left({\boldsymbol{\alpha}}_g+{\mathbf{u}}_{ig}\right)\right)\\&-\frac{1}{2}{\mathbf{u}}_{ig}^{\prime}{\boldsymbol{\varphi}}_g^{-1}{\mathbf{u}}_{ig} \end{array} $$

where

12 $$ {w}_{ig}^{(t)}=\frac{\pi_g^{(t)}f\left({\mathbf{x}}_i\Big|{\theta}_g^{(t)}\right)}{{\displaystyle \sum_{g=1}^G{\pi}_g^{(t)}f\left({\mathbf{x}}_i\Big|{\theta}_g^{(t)}\right)}} $$

denotes the posterior probability that **x**_*i*_ arises from the *g-th* component. In the M-step of the algorithm, we maximize *Q* ^(*t*)^(⋅) with respect to *θ*_*g*_ and *π*_*g*_. Using the conditional expectation of the sufficient statistics. Since **u**_*ik*_' **u**_*ik*_ and (**x**_*ik*_ − **Vu**_*ik*_), we obtain the following estimates for *g* = 1, …, *G*:

13 $$ {\widehat{\pi}}_g^{(t)}=\frac{{\displaystyle \sum_i}{w}_{ig}^{(t)}}{n} $$

14 $$ {\boldsymbol{\upalpha}}_g^{\left(t+1\right)}={\boldsymbol{\upalpha}}_g^{(t)}+\frac{diag\left({\boldsymbol{\upsigma}}_g^{2(t)}\right){\left(\mathbf{V}\hbox{'}\mathbf{V}\right)}^{-1}\mathbf{V}\hbox{'}{\left(\mathbf{V}{\boldsymbol{\Phi}}_g^{(t)}\mathbf{V}\hbox{'}+ diag\left(\mathbf{V}{\boldsymbol{\upsigma}}_g^{2(t)}\right)\right)}^{-1}{\displaystyle \sum_i{w}_{ig}^{(t)}\left[\left({\mathbf{x}}_i-\mathbf{V}{\boldsymbol{\upalpha}}_g^{(t)}\right)\right]}}{{\displaystyle \sum_i{w}_{ig}^{(t)}}} $$

15 $$ {\sigma}_{gh}^{2\left(t+1\right)}=\frac{1}{{\mathbf{V}}_h\hbox{'}{\mathbf{V}}_h{\displaystyle \sum_i{w}_{ig}^{(t)}}}{\displaystyle \sum_i{w}_{ig}^{(t)}\left[\left({\mathbf{x}}_i-\mathbf{V}\left({\boldsymbol{\upalpha}}_g^{(t)}-{\mathbf{u}}_{ig}^{(t)}\right)\right)\ \hbox{'}{\left( diag\left(\mathbf{V}{\boldsymbol{\upsigma}}_g^{2(t)}\right)\right)}^{-1}\left({\mathbf{x}}_i-\mathbf{V}\left({\boldsymbol{\upalpha}}_g^{(t)}-{\mathbf{u}}_{ig}^{(t)}\right)\right)\right]+{\gamma}_g^{(t)}} $$

where **V**_*h*_ is the *h*-th column vector of **V**(*h* = 1, 2), **u**_*ig*_^(*t*)^ is a flexible estimator of **u**_*ig*_ based on its posterior mean conditional on the observed data, given by

16 $$ E\left({\mathbf{u}}_{ig}\Big|\mathbf{x}\right)={w}_{ig}^{(t)}{\boldsymbol{\Phi}}_g^{(t)}\mathbf{V}\hbox{'}{\left(\mathbf{V}{\boldsymbol{\Phi}}_g^{(t)}\mathbf{V}\hbox{'}+ diag\left(\mathbf{V}{\boldsymbol{\upsigma}}_g^{2(t)}\right)\right)}^{-1}\left({\mathbf{x}}_i-\mathbf{V}{\boldsymbol{\upalpha}}_g^{(t)}\right) $$

and *γ*_*g*_^(*t*)^ corresponds to the trace of the current conditional covariance matrix cov(**e**_*ig*_|**x**), and, finally,

17 $$ \begin{array}{ll}{\boldsymbol{\Phi}}_g^{\left(t+1\right)}=\hfill & \frac{{\displaystyle \sum_i{w}_{ig}^{(t)}{\boldsymbol{\Phi}}_g^{(t)}\mathbf{V}\hbox{'}}{\left(\mathbf{V}{\boldsymbol{\Phi}}_g^{(t)}\mathbf{V}\hbox{'}+ diag\left(\mathbf{V}{\boldsymbol{\upsigma}}_g^{2(t)}\right)\right)}^{-1}\left({\mathbf{x}}_i-\mathbf{V}{\boldsymbol{\upalpha}}_g^{(t)}\right)\left({\mathbf{x}}_i-\mathbf{V}{\boldsymbol{\upalpha}}_g^{(t)}\right)\ \hbox{'}{\left(\mathbf{V}{\boldsymbol{\Phi}}_g^{(t)}\mathbf{V}\hbox{'}+ diag\left(\mathbf{V}{\boldsymbol{\upsigma}}_g^{2(t)}\right)\right)}^{-1}\mathbf{V}{\boldsymbol{\Phi}}_g^{(t)}}{{\displaystyle \sum_i{w}_{ig}^{(t)}}}\hfill \\ {}\hfill & +\left[\mathbf{I}-{\boldsymbol{\Phi}}_g^{(t)}\mathbf{V}\hbox{'}{\left(\mathbf{V}{\boldsymbol{\Phi}}_g^{(t)}\mathbf{V}\hbox{'}+ diag\left(\mathbf{V}{\boldsymbol{\upsigma}}_g^{2(t)}\right)\right)}^{-1}\mathbf{V}\right]{\boldsymbol{\Phi}}_g^{(t)}\hfill \end{array} $$

The EM algorithm to obtain the ML parameter estimates given the number of classes *G* has been implemented by using an adaptation of the Emmixwire program for the R environment; the original script is freely available at http://www.maths.uq.edu.au/~gjm/mix_soft/EMMIX-WIRE/index.html. The number of components has to be chosen according to the BIC.

## Abbreviations

IGF-1: Insulin-like growth factor 1
FMM: Finite mixture model
FMLMM: Finite mixture of linear mixed models
KDE: Kernel density estimation
AIC: Akaike Information criterion
BIC: Bayesian Information Criterion
MAP: Maximum a posteriori
Sk. muscle: Skeletal muscle
UTR: Untranslated region
Chr: Chromosome
